# The Complex Contributions of Genetics and Nutrition to Immunity in *Drosophila melanogaster*

**DOI:** 10.1371/journal.pgen.1005030

**Published:** 2015-03-12

**Authors:** Robert L. Unckless, Susan M. Rottschaefer, Brian P. Lazzaro

**Affiliations:** Department of Entomology, Cornell University, Ithaca, New York, United States of America; University of Pennsylvania School of Medicine, UNITED STATES

## Abstract

Both malnutrition and undernutrition can lead to compromised immune defense in a diversity of animals, and “nutritional immunology” has been suggested as a means of understanding immunity and determining strategies for fighting infection. The genetic basis for the effects of diet on immunity, however, has been largely unknown. In the present study, we have conducted genome-wide association mapping in *Drosophila melanogaster* to identify the genetic basis for individual variation in resistance, and for variation in immunological sensitivity to diet (genotype-by-environment interaction, or GxE). *D*. *melanogaster* were reared for several generations on either high-glucose or low-glucose diets and then infected with *Providencia rettgeri*, a natural bacterial pathogen of *D*. *melanogaster*. Systemic pathogen load was measured at the peak of infection intensity, and several indicators of nutritional status were taken from uninfected flies reared on each diet. We find that dietary glucose level significantly alters the quality of immune defense, with elevated dietary glucose resulting in higher pathogen loads. The quality of immune defense is genetically variable within the sampled population, and we find genetic variation for immunological sensitivity to dietary glucose (genotype-by-diet interaction). Immune defense was genetically correlated with indicators of metabolic status in flies reared on the high-glucose diet, and we identified multiple genes that explain variation in immune defense, including several that have not been previously implicated in immune response but which are confirmed to alter pathogen load after RNAi knockdown. Our findings emphasize the importance of dietary composition to immune defense and reveal genes outside the conventional “immune system” that can be important in determining susceptibility to infection. Functional variation in these genes is segregating in a natural population, providing the substrate for evolutionary response to pathogen pressure in the context of nutritional environment.

## Author Summary

Previous studies have indicated that dietary nutrition influences immune defense in a variety of animals, but the mechanistic and genetic basis for that influence is largely unknown. We use the model insect *Drosophila melanogaster* to conduct an unbiased genome-wide mapping study to identify genes responsible for variation in resistance to bacterial infection after rearing on either high-glucose or low-glucose diets. We find the flies are universally more susceptible to infection when they are reared on the high-glucose diet than when they are reared on the low-glucose diet, and that metabolite levels genetically correlate with quality of immune defense after rearing on the high-glucose diet. We identify several genes that contribute to variation in defense quality on both diets, most of which are not traditionally thought of as part of the immune system. The genetic variation we observe can be important for evolved responses to pathogen pressure, although the effectiveness of natural selection will be partially determined by the host nutritional state.

## Introduction

There is strong intuition that dietary nutrition affects the quality of immune defense, and this intuition is well supported scientifically. Starvation increases susceptibility to infection in insects as well as humans [1,2], and specific dietary components such as vitamins, carbohydrates, and proteins have been implicated in shaping immunity to bacterial infection [3–7]. Elevated dietary protein relative to sugar increases standing levels of immune activity in *Drosophila melanogaster* [8], and diets deficient in protein increase susceptibility to infection by *Salmonella typhimurium* in mice [6]. Nutrition alters development in ways that may have immunological import [9–11], and insects and other animals alter their feeding behavior in response to infection [12,13]. There is growing evidence that the ratio of protein to carbohydrates (P:C) in the diet may specifically influence several life history traits[11,14–18], including some that may predict resistance to infection. For example, the African army worm *Spodoptera exempta* becomes more susceptible to infection by the bacterium *Bacillus subtilis* when supplied with diets high in sugar relative to protein, and infected caterpillars will actively choose to eat diets higher in protein without increasing sugar intake [13]. These and other such observations have led to the suggestion that “nutritional immunology” should be employed to identify ideal dietary compositions for the combat of infection [4]. However, despite the increasingly clear impact of diet on resistance to infection, we have remarkably little insight into *how* nutrition alters infection outcomes, and whether or why individuals in natural populations differ genetically in their immunological response to diet.

Natural populations are rife with genetic variation for traits that determine health and evolutionary fitness, and both human and *Drosophila* populations are genetically variable for the ability to fight bacterial infection [19,20]. Such variation may occur in intuitively evident genes, such as those that make up the immune system [21,22], but phenotypically important variation may also map to less obvious genes that shape host physiological context. Even traits that have strong genetic determination can be influenced by the environment, including the availability of nutrition [23,24]. Importantly, different genotypes can vary in their susceptibility to environmental influence, resulting in traits that are determined by the interaction between genotype and environment (GxE) [25]. In very few cases, however, have the genes underlying sensitivity to environment been determined, and it is indeed difficult to predict *a priori* what the genes for environmental sensitivity might be. The genetic variation that controls both direct trait determination as well as that that controls environmentally influenced phenotypic variation are critically important to the health and evolutionary potential of populations.

We have previously used candidate-gene based approaches to map the genetic basis for variation in *Drosophila melanogaster* resistance to bacterial infection [26–28]. These studies were successful in identifying naturally occurring alleles that shape defense quality, but they focused exclusively on genes in the immune system. While we may expect diet to shape resistance to infection, we have no particular expectation that the effects of diet act through the canonical immune system (*i*.*e*. Toll and IMD pathways [29,30]). Dietary composition has widespread metabolic and developmental consequences, and these consequences vary quantitatively and qualitatively among genetically diverse *Drosophila* [31]. There is evidence for crosstalk between metabolic signaling pathways such as insulin-like signaling and canonical immune pathways in *Drosophila*, both during development and in the initiation of an immune response [32–36]. Thus, it is plausible to imagine that the immunological effect of diet, and especially genetic variation in immunological response to diet (genotype-by-diet interaction), could be controlled by genes outside of what is typically conceived to be the “immune system.”

In the present study, we conduct an unbiased genome-wide association study to identify genes that shape variation in resistance to bacterial infection among *D*. *melanogaster* reared on either a high glucose or low glucose diet. Specifically, we deliver experimental infections with the bacterium *Providencia rettgeri* and measure systemic pathogen load 24-hours post infection. This time point both provides a robust estimate of infection intensity [37] and correlates strongly with risk of mortality [38]. Throughout the manuscript we will refer to pathogen load as “resistance” or “immune defense”. We find that flies reared on a high glucose diet harbor significantly higher pathogen loads and substantially altered metabolite levels, including elevated free glucose, glycogen and triglycerides. Although there is considerable natural genetic variation for resistance to infection on both diets, resistance is generally well correlated across the two diets. Nonetheless, we find evidence of genotype-by-environment interactions determining immune defense, as well as metabolic alterations that correlate genetically with resistance in flies reared on the high glucose diet. We are able to map and validate several genes that contribute to variation in resistance in both diet-independent and diet-dependent manners. Importantly, most of these are not typically considered part of the canonical immune system.

## Results

### Resistance to infection varies genetically and across diets

**Fig 1:**
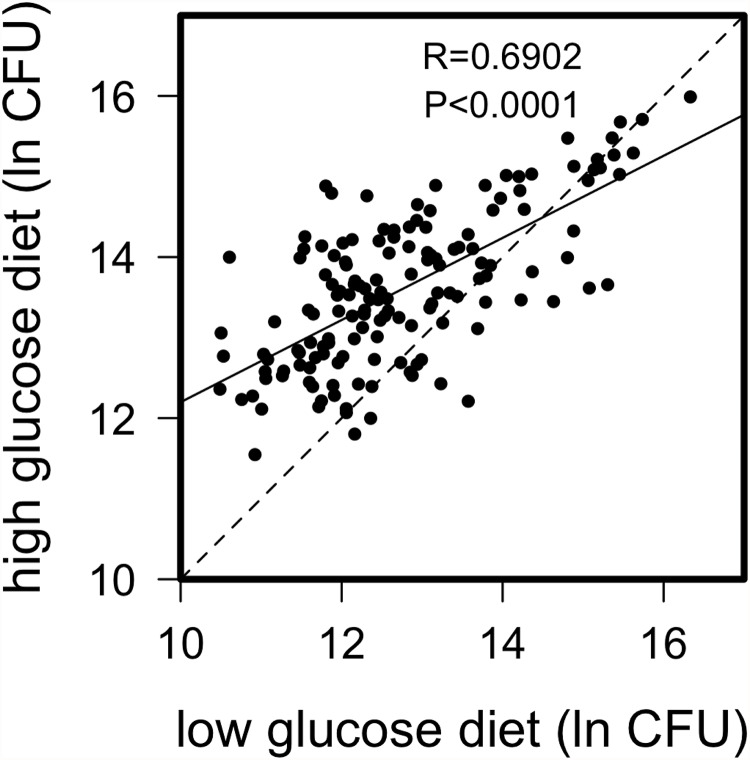
Correlation of natural log bacterial load (CFU) 24-hours post infection for DGRP lines raised on high glucose and low glucose diets. Dashed line represents 1 to 1 relationship; solid line from regression analysis. There is strong correlation across diets, but several lines appear to perform disproportionately poorly (*i*.*e*. carry high bacterial load) on the high glucose diet. A natural log value of 10 corresponds to about 2.2x10^4^ bacteria, 12 corresponds to about 1.6x10^5^ bacteria, 14 corresponds to 1.2x10^6^ bacteria and 16 corresponds to 8.9 x10^6^ bacteria.

We found considerable natural genetic variation for immune defense segregating within the *Drosophila* Genetic Reference Panel (DGRP), where the quality of defense is defined as the ability to limit pathogen proliferation. We infected male flies from 172 of the complete genome-sequenced lines [39] with the Gram-negative bacterium *Providencia rettgeri* after rearing on either a high glucose or low glucose diet in a replicated block design (see Methods), then measured systemic pathogen load 24 hours later. Pathogen load was significantly predicted by line genotype and diet (Table 1; S1 Fig, *p* < 10^-4^ for both) as well as by a genotype-by-diet interaction (*p* = 0.0016), indicating that genotypes differ in their immunological sensitivity to dietary glucose. Nonetheless, pathogen load was highly correlated across the two diets (Pearson *r* = 0.69, *p* < 10^-4^; Fig. 1), indicating a strong main effect of genotype on immune performance. On average, flies reared on the high-glucose diet sustained systemic pathogen loads approximately 2.4 times higher than those of flies reared on the low-glucose diet.

**Table 1:** ANOVA results for phenotypes measured F value/Z value (*p-value*) showing significant line, diet and line by diet interaction effects for most phenotypes.

Factor	Ln CFU	Glucose	Protein	Triglyceride	Glycerol	Glycogen	Weight
*Wolb*	2.47 (0.015)	0.03 (0.85)	4.04 (0.046)	0.10 (0.75)	0.74 (0.39)	0.25 (0.62)	0.15 (0.70)
Diet	6.38 (<0.0001)	95.53 (<0.0001)	15.42 (<0.0001)	121.35 (<0.0001)	0.01 (0.93)	2.72 (0.0066)	46.92 (<0.0001)
Line(*Wolb*)	6.83 (<0.0001)	1.30 (0.097)	3.39 (0.0003)	4.49 (<0.0001)	5.35 (<0.0001)	5.76 (<0.0001)	6.93 (<0.0001)
Diet*Line(*Wolb*)	2.95 (0.0016)	5.07 (<0.0001)	0.205 (0.0057)	0.78 (0.21)	0.04 (0.48)	1.09 (0.013)	2.05 (0.020)
Infector	47.33 (<0.0001)	NA	NA	NA	NA	NA	NA
Plater	3.74 (0.054)	NA	NA	NA	NA	NA	NA
Block(Diet)	22.45 (<0.0001)	16.94 (<0.0001)	7.37 (<0.0001)	3.54 (0.0037)	24.9 (<0.0001)	16.1 (<0.0001)	17.91 (<0.0001)

*Wolb* = *Wolbachia* infection status

### Diet and genotype influence nutritional status

**Fig 2:**
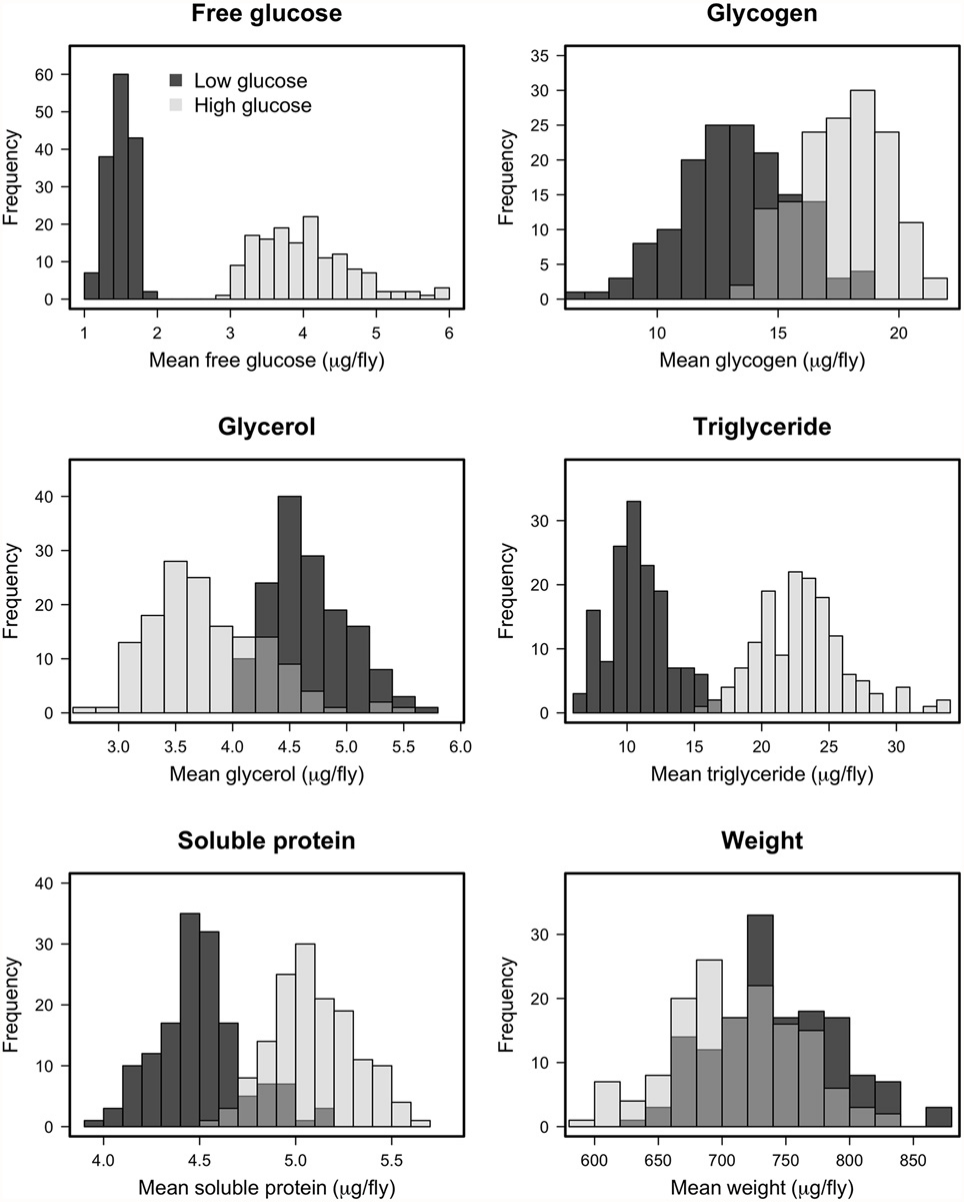
Histograms of estimated line means for nutritional indices for DGRP lines reared on high glucose and low glucose diets. The differences in distributions on the high glucose and low glucose diets were highly significant (*p <* 10^-4^) in all cases, supporting the assertion that diet significantly alters the metabolic state of the fly.

**Fig 3:**
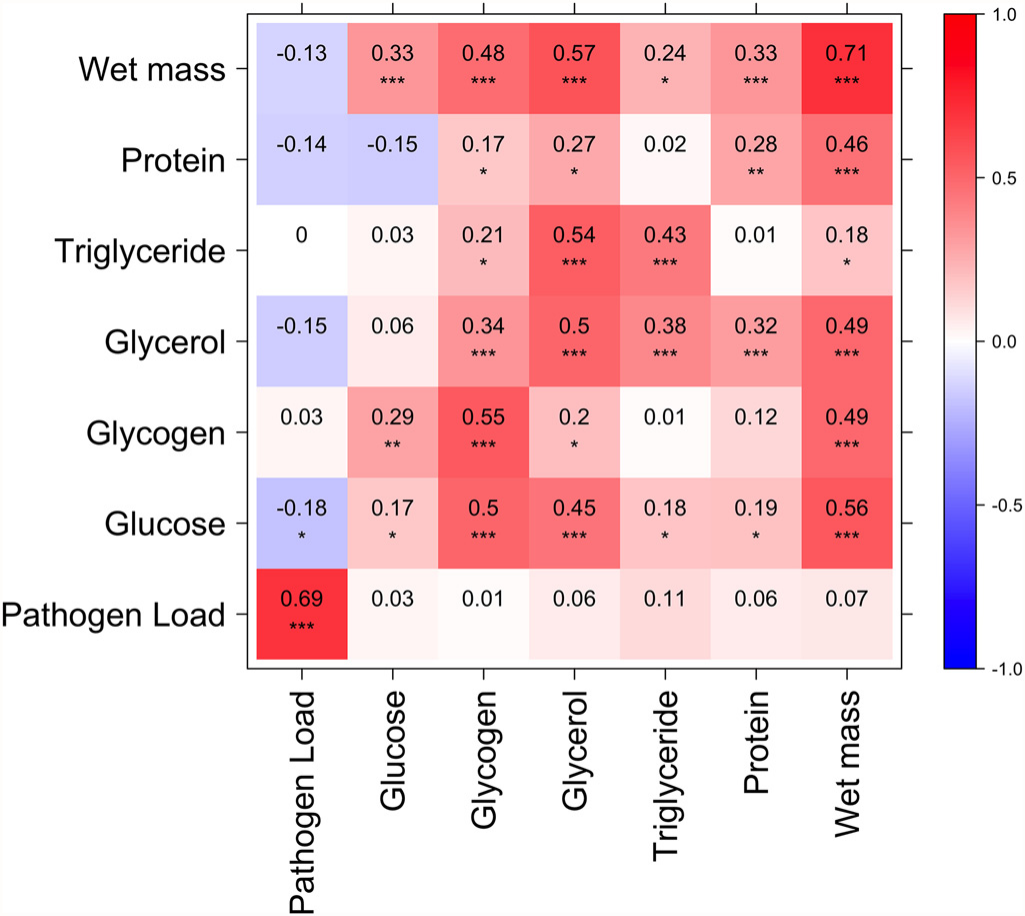
Correlation between nutritional indices and immune defense (Ln CFU per fly). Diagonal represents correlation for each index between high and low glucose diets; above diagonal is correlation among indices on the high glucose diet; below diagonal is correlation among indices on the low glucose diet. *p*<0.0001***, *p*<0.001**, *p*<0.05*. Several nutritional indices are correlated with each other but only glucose on the high glucose diet is significantly (negatively) correlated with immune defense.

We measured several indices of nutritional status in each *Drosophila* line after rearing on the high-glucose and low-glucose diets because we predicted that specific metabolite profiles might be associated with changes in immunity. We measured free glucose, glycogen stores, total triglycerides, free glycerol, soluble protein, and wet mass, as these provide an overall picture of an individual’s nutritional status. The Nutritional Indices (NIs) showed predictable responses to diet. For example, levels of glucose, glycogen, and triglycerides were substantially elevated by rearing on the high-glucose diet (Fig. 2; *p* < 10^-4^ in all cases), although wet weight and free glycerol were significantly reduced by rearing on high glucose (Fig. 2; *p* < 10^-4^ in both cases). The lines exhibited highly significant genetic variation for all NIs after rearing on either diet (*p* < 10^-4^ in all cases; Table 2). Each NI was significantly genetically correlated across diets (Fig. 3), indicating strong genetic determination of NIs regardless of diet. Surprisingly, only wet mass, glycogen and free glucose showed strong genotype-by-diet interactions (Table 1).

**Table 2:** Effect of genetic line in determining traits on each diet (Z-values—all P-values are less than 0.0001).

Diet	CFU	Glucose	Protein	Triglyceride	Glycerol	Glycogen	Wet mass
Low glucose	23.31	11.36	12.16	12.03	12.05	11.23	12.13
High glucose	22.78	8.16	11.96	8.10	11.93	8.11	11.93

All phenotypes show strong line effects on when data from the two diets are considered separately.

### Resistance to infection is correlated with nutritional status and other phenotypes

Since we found that increasing dietary glucose resulted in increased pathogen load as well as alteration of metabolic profile, we asked whether metabolic profile correlated with pathogen load across genotypes. The only NI that correlated with pathogen load was free glucose, which was slightly negatively correlated with *P*. *rettgeri* load on the high-glucose diet (Pearson’s *r* = -0.18, *p* = 0.033). This is somewhat surprising given that the general effect of increased dietary glucose is both elevated blood glucose and an increase in pathogen load, and may indicate that variation in pathogen load is associated with rates of conversion between molecules.

We hypothesized that genetic variation might shape the relationship between overall metabolic state and immune defense and that our nutritional indices might give more information about the overall metabolic status of the fly when considered in aggregate. We therefore performed a principal component analysis and tested whether the primary principal components (PCs) for each diet correlated with immune defense quality. The top five PCs summarizing the NIs on each diet each explain 8–41% of the total variance in nutritional state, with loadings of each NI given in Table 3. None of the metabolic PCs correlated with pathogen load on the low-glucose diet. The fourth PC on the high-glucose diet was significantly correlated with pathogen load (Pearson’s *r* = -0.27, *p* = 0.001; Table 3). This PC, which explains 11% of the total variance, is heavily positively loaded with free glucose (0.58) and soluble protein (0.36) and is negatively loaded with glycogen stores (-0.71), consistent with the observation that free glucose alone is negatively correlated with pathogen load. This is the only PC where free glucose and glycogen stores load in opposite directions, possibly indicating the rate of conversion between dietary glucose to glycogen. PC1 trends toward negative correlation with pathogen load on the high-glucose diet (Pearson’s *r* = -0.15; *p* = 0.06). This PC explains 37% of the variance and is positively loaded with all NIs, and presumably reflects overall fly mass although mass itself does not correlate with pathogen load (Fig. 3).

**Table 3:** Principal component analysis of nutritional phenotypes with data from each diet considered separately.

Treatment	Component	PC1	PC2	PC3	PC4	PC5
Low glucose	St. dev.	1.42	1.05	0.98	0.72	0.64
	Variance prop.	0.41	0.22	0.19	0.10	0.08
	Protein	-0.33	0.06	0.86	0.35	-0.14
	Glucose	-0.56	0.24	-0.22	-0.32	-0.69
	Triglyceride	-0.31	-0.72	-0.31	0.52	-0.14
	Glycerol	-0.55	-0.31	0.13	-0.53	0.55
	Glycogen	-0.42	0.57	-0.31	0.46	0.43
	Load R	-0.09	-0.10	-0.04	0.01	-0.03
High glucose	St. dev.	1.36	1.10	0.98	0.75	0.63
	Variance prop.	0.37	0.24	0.19	0.11	0.08
	Protein	0.27	0.54	0.64	0.36	-0.30
	Glucose	0.18	-0.76	0.22	0.58	-0.08
	Triglyceride	0.52	0.06	-0.59	0.06	-0.61
	Glycerol	0.62	0.17	-0.16	0.19	0.72
	Glycogen	0.49	-0.30	0.41	-0.71	-0.07
	Load correlation	-0.15	-0.02	-0.08	-0.27**	-0.09

*P<0.05;

**P<0.01

When we compared our data to previously published DGRP phenotype data [39], we found correlations between our NIs and three metabolism-related traits: starvation resistance, chill coma recovery, and startle response. Starvation resistance, as determined by Mackay *et al*. [20], is positively correlated with all of our NIs except soluble protein with correlation coefficients ranging from 0.169 (Table 4, *p* = 0.044) to 0.388 (*p* < 10^-4^). Overall, genotypes with greater energy reserves were better able to withstand the stress of starvation: measures of wet mass, soluble protein, and glycogen stores were significantly negatively correlated with time to recovery from chill coma as measured by Mackay *et al*. (chill coma recovery correlated with wet mass: *r* = -0.235, *p* = 0.005; soluble protein: *r* = -0.248, *p* = 0.003; glycogen: *r* = -0.20, *p* = 0.016). Our measures of free glucose levels and total triglycerides were weakly correlated with Mackay *et al*.’s measure of startle response (Pearson’s *r* < 0.20 and *p* < 0.05 for each). This consistency in related phenotypic measures and relationships across lab groups indicates the genetic robustness of the phenotypes.

**Table 4:** Correlation coefficients *(r)* of phenotypes measured in this study to those of Mackay *et al*. (2012) show several significant correlations.

Our Phenotype	Startle Response	Chill Coma	Stress Resistance
Weight	0.016	-0.235**	0.262**
Bacterial Load	0.101	-0.024	0.079
Soluble Protein	-0.074	-0.248**	-0.085
Glucose	0.169*	-0.12	0.254**
Glycogen	0.144	-0.2*	0.364***
Glycerol	0.003	-0.085	0.169*
Triglyceride	0.168*	0.045	0.388***

*P<0.05

**P<0.01

***P<0.001

The bacterial endosymbiont *Wolbachia pipientis* has been shown to confer protection against RNA viruses in *Drosophila* [40,41], but previous experiments have not uncovered any protective benefit of *Wolbachia* against secondary bacterial infection [42,43]. Richardson *et al*. [44] determined that 52% of the lines in the DGRP are infected with *Wolbachia*, and we find *Wolbachia* status to be a weakly significant predictor of *P*. *rettgeri* load on both diets (S2 Fig, low glucose: *p =* 0.0361; high glucose: *p =* 0.0327; data from both diets combined: *p =* 0.014), with lower average bacterial loads in the *Wolbachia-*infected lines than in the *Wolbachia-*uninfected lines.

### Genome-wide association mapping of resistance to infection

Because the complete genomes have been sequenced for every line in the DGRP, we were able to conduct unbiased genome-wide association mapping for each of our measured phenotypes. We used mixed effect linear models to identify genetic polymorphisms that predict systemic pathogen load. Using a significance criterion of 10^-6^ we identified seven single nucleotide polymorphisms (SNPs) in six genes that associate with variation in pathogen load on the high-glucose diet, 11 SNPs in 9 genes that associate with load on the low-glucose diet, and 19 SNPs in 12 genes that associate with pathogen load when the data from both diets is pooled (Table 5; S3 and S4 Figs). This significance threshold corresponds to a false discovery rate of 5–10% (depending on the phenotypic distribution of the particular trait being evaluated and the details of the analytical model) and provided a reasonable number of SNPs for further characterization. Several of the mapped SNPs were common to multiple analyses. Overall, we mapped SNPs in the genes *crinkled*, *defective proboscis extension response 6*, *diptericin*, *elk*, *fruitless*, *kinesin heavy chain 73*, *multiplexin*, *Scr64B*, *sema-1a*, *tout velu*/CG12869, *CG42524*, *CG7991*, *CG4835* and *CG15544*. We additionally mapped SNPs to 2 distinct regions annotated to encode small, nontranslated RNAs, potentially revealing variation for more complex regulation of the immune system [45].

**Table 5:** Significant SNPs (P<10^-6^) from genome-wide association study for immune defense against *Providencia rettgeri* infection with data from the high glucose diet (*p*_*high*_), low glucose diet (*p*_*los*_) and when data from both diets are combined (*p*_*pooled*_).

SNP	gene	class	A1	A2	MAF	*p*_*high*_	*p*_*low*_	*p*_*pooled*_
2L.8630728	Sema-1a*	intron	T	C	0.45	1.42E-06	0.0012	1.87E-05
2L.13072327	s2_48__2_1156898	snRNA	T	G	0.45	9.30E-07	4.00E-06	9.79E-08
2L.15045678	ck	intron	T	A	0.38	1.94E-07	0.0075	4.29E-05
2L.7632178	*none annotated*		G	A	0.04	1.30E-06	5.03E-07	9.82E-08
2R.10477114	ttv/CG12869	1594/1692	T	A	0.03	8.87E-05	7.65E-07	1.48E-06
2R.11405165	Khc-73	3′ UTR	T	C	0.17	4.92E-07	7.15E-08	5.48E-08
2R.11413959	Khc-73	syn.	T	C	0.17	1.98E-05	4.46E-07	3.10E-07
2R.11502756	CG42524	nonsyn.	A	T	0.1	6.00E-05	2.16E-05	9.84E-07
2R.11502761	CG42524	nonsyn.	T	A	0.1	6.00E-05	2.16E-05	9.84E-07
2R.13779189	elk	intron	T	A	0.15	4.49E-05	6.75E-07	5.31E-06
2R.14753586	Dpt	syn.	G	A	0.14	9.03E-07	2.39E-07	7.04E-08
2R.14753589	Dpt	nonsyn.	C	A	0.14	9.03E-07	2.39E-07	7.04E-08
3L.10039434	dpr6	intron	G	A	0.05	3.21E-05	9.54E-08	3.51E-07
3L.10334296	*none annotated*		G	T	0.03	1.72E-06	6.05E-07	5.40E-07
3L.10413672	V085_8048390	snRNA	G	T	0.07	3.12E-05	1.21E-06	9.63E-07
3L.1704443	CG7991	intron	G	A	0.21	1.42E-05	2.13E-06	3.25E-07
3L.4603286	Src64B	intron	G	A	0.08	2.50E-07	1.41E-07	3.34E-09
3L.5468191	CG4835	up(4899)	A	C	0.03	3.90E-05	7.94E-07	6.67E-07
3L.7005402	mp	intron	T	C	0.44	7.59E-06	4.46E-06	3.90E-07
3L.7005411	mp	intron	C	T	0.44	1.00E-05	4.12E-06	4.45E-07
3L.7005473	mp	intron	A	T	0.5	2.25E-07	8.64E-05	8.78E-07
3R.14298230	fru	intron	C	T	0.05	6.75E-06	1.50E-06	7.42E-07
3R.26670883	CG15544	dn(3127)	T	C	0.03	3.97E-05	9.86E-07	1.88E-07

*misses nominal significance threshold, but included since appears potentially diet-specific

SNP = position in genome (chromomsome.position); gene = gene symbol ID; class = type of SNP; A1 = allele 1 ID; A2 = allele 2 ID; MAF = minor allele (A2) frequency.

Our mapped SNPs are highly enriched for lying within or adjacent to genes, with 21 of the total 23 (91.3%) lying within 5 kb of an annotated gene (Table 5). In contrast, only 55% of SNPs genome-wide lie within 5 kb of a known gene. Of our 5 mapped SNPs within gene coding regions, 2 are synonymous and 3 are nonsynonymous, again in stark contrast to the genome average, for which there are approximately 2.7 synonymous polymorphisms for every nonsynonymous polymorphism [46]. One of the two synonymous variants we mapped is in perfect linkage disequilibrium with an amino-acid-altering SNP in *Diptericin*. The other is in perfect disequilibrium with a 3′ UTR variant of *kinesin heavy chain 73*. Thus, both of our mapped synonymous SNPs can be considered to be redundant with more plausibly functional SNPs. We used RNAi to knock down 13 of the mapped genes, 9 of which resulted in significantly altered pathogen load either on a standard diet or in a diet-specific manner (S5 Fig, S1 Table). In contrast, only one of five control genes chosen by virtue of physical proximity to mapped genes yielded an altered bacterial load phenotype after RNAi knockdown.

**Fig 4:**
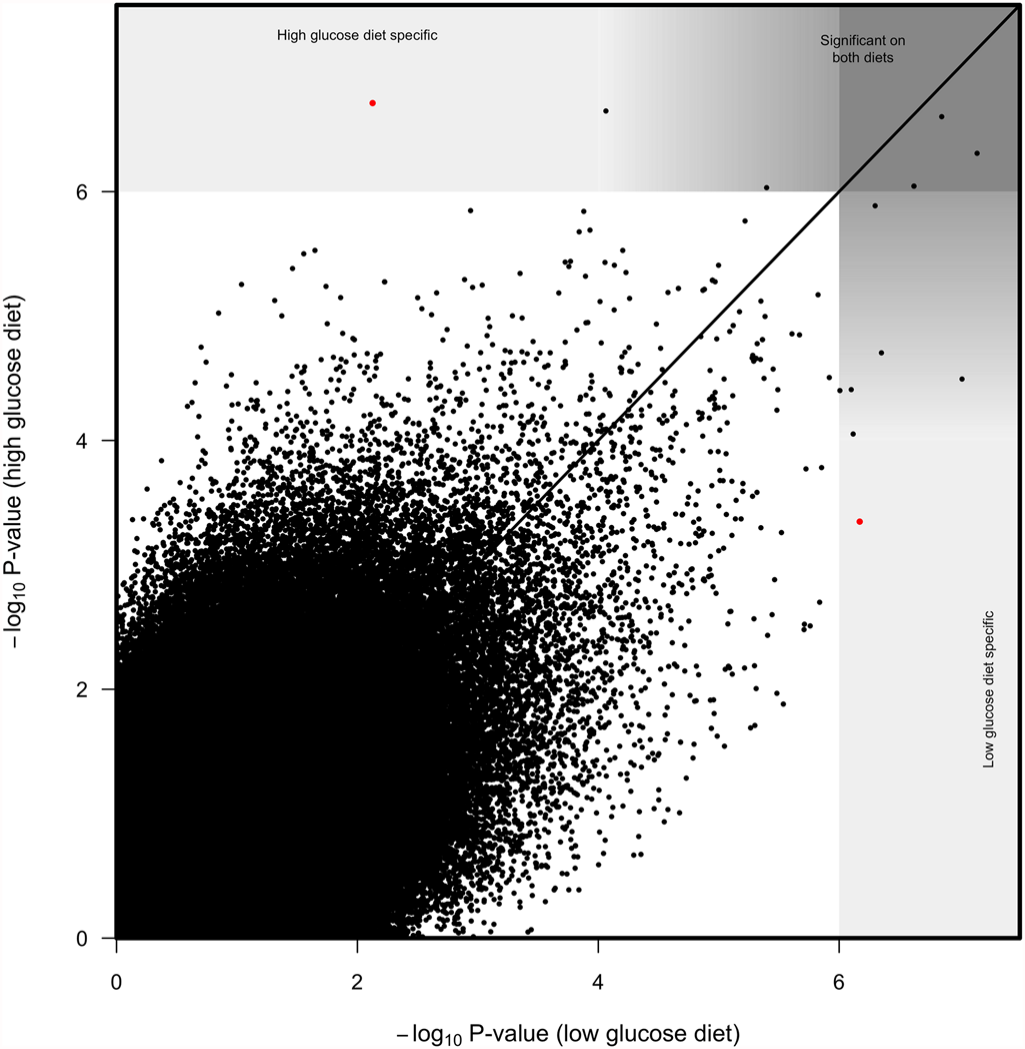
Correlation between SNP log_10_ *p-*values from genome wide associations on high glucose diet and low glucose diet. Solid line represents 1 to 1 value. While most significant SNPs were significant on both diets, we considered SNPs with *p*<10^-6^ on one diet and *p*>10^-4^ on the other diet to be diet specific.

To identify SNPs that have strongly diet-dependent effects on immunity, we first considered SNPs that had significant effects (*p* < 10^-6^) on one diet but not on the other (*p* > 10^-4^; Fig. 4). Only a few SNPs meet this criterion. One SNP in *crinkled* (2L.15045678) was significantly associated with variation in immunity on the high glucose diet (*p* = 1.94 x 10^-7^) but not on the low glucose diet (*p* = 0.0074). A SNP in *Sema-1a* (2L.8630728) was very on the brink of significance on the high glucose diet (*p* = 1.42 x 10^-6^) and nowhere near our significance threshold on the low glucose diet (*p* = 0.001). Reciprocally, one SNP in *elk* (2R.13779189) was significant on the low glucose diet (*p* = 6.75 x 10^-7^) but not on the high glucose diet (*p* = 4.49 x 10^-4^). All SNPs with *p <*10^-4^ on either diet were significant at *p* < 10^-6^ when the data from both diets were pooled.

Our second approach to finding genes with significant diet-dependent effects was to pool the data from both diets and evaluate the SNP-by-diet interaction in a second GWAS analysis. While this approach resulted in a somewhat liberal inflation in P-values (S4b Fig), it revealed SNPs in several genes has having diet-dependent effects at a nominal threshold of *p* < 10^-6^. Of the genes mapped with this second approach, we chose *TepII*, *gprk2*, and *similar* to test by RNAi, and confirmed the importance of these genes on suppression of *P*. *rettgeri* proliferation (see below).

To determine whether any gene function categories were enriched in our set of significantly mapped SNPs, we performed a GO enrichment analysis using GOWINDA [47], which corrects for gene size, on the reduced GO category list defined by GO Slim [48]. Because so few SNPs mapped significantly at our cutoff of *p*<10^-6^, we performed the GO analysis at a significance threshold of *p*<10^-5^. Categories related to immunity and metabolism were among the most enriched, but no functional categories were significantly enriched after multiple correction (S2 Table). GO analysis of GWAS results implicitly assumes a quantitative genetic model where many genes in every relevant functional process each contribute small but significant effects on the overall phenotype. We have no evidence that this is an appropriate conceptual model for our defense phenotype, so we did not pursue the GO analysis further.

We performed genome-wide association mapping of each of the nutritional indices, yielding several hits in or near genes with reasonable links to metabolic status [49]. We found no overlap between SNPs significantly associated with variation in the NIs and those significantly associated with variation in immune defense. The genetic basis for altered nutritional status in response to diet will be the subject of an independent paper [49].

### Characterization and RNAi knockdown of genes mapped for resistance to infection

***Diptericin*.** *Diptericin* is a antimicrobial peptide that is produced in response to DAP-type peptidoglycan that makes up the cell walls of Gram-negative bacteria such as *P*. *rettgeri* [50,51]. Two SNPs in perfect linkage disequilibrium (2R.14753586—synonymous, 2R.14753589—nonsynonymous) are significantly associated with variable suppression of *P*. *rettgeri* infection in flies reared on both diets (*p* = 9.03 x 10^-7^ for each SNP on high glucose and *p* = 2.93 x 10^-7^ on low glucose) as well as when data from the two diets are pooled (*p* = 7.04 x 10^-08^ for each SNP). While it might seem intuitive that an antibacterial peptide gene would map in an immunity screen, this result was surprising as we have not identified any marked effect of *Diptericin* in previous association studies using other Gram-negative bacterial infections [19,27]. Indeed, it is generally believed that there is enough redundancy in AMPs that mutations in a single peptide would have little effect on organism-level immunity [e.g. 52]. The nonsynonymous SNP (2R.14753589) results in a serine versus arginine polymorphism segregating in the population. In the DGRP, the more resistant serine allele is carried by 82% of lines and the more permissive arginine by 14% of lines (4% of lines are heterozygous at the SNP). Two of the DGRP lines are homozygous for a premature stop codon in *Diptericin* at position 2R.14753502. While this stop codon did reach not our minor allele frequency threshold for consideration in the study, we thought it was notable that two lines carrying the premature termination exhibited the absolute highest bacterial loads across the entire DGRP mapping panel. Both of these lines carried the higher-resistance serine variant at 2R.14753589, thereby slightly decreasing the statistical significance of the independent contrast between the serine and arginine variants. If these two lines are excluded from the analysis, the P-value for 2R.14753589 is 4.43x10^-9^. Interestingly, we found that serine and arginine are also segregating in *Drosophila simulans* through an independent mutation at the same codon, suggesting the possibility of convergent balancing selection (Unckless *et al*. in prep.; see Discussion).

***Multiplexin.*** *Multiplexin* (*mp*) encodes a collagen protein. *Multiplexin* is a huge gene (55 kb) with 15 annotated transcripts. Annotated molecular functions include carbohydrate binding and motor neuron axon guidance [53]. Loss-of-function mutants have smaller larval fat bodies than wild-type flies [54], which may be relevant since the fat body is the primary tissue that drives systemic immunity to bacterial infection. Three intronic SNPs in *mp* are significantly associated with variation in *P*. *rettgeri* load in flies reared on either the high glucose (*p* = 2.25 x 10^-7^) or low glucose (*p* = 4.12 x 10^-6^) diet, as well as when data from both diets are pooled (*p =* 3.9 x 10^-7^). Ubiquitous RNAi knockdown of *mp* resulted in significantly decreased *P*. *rettgeri* load after infection relative to controls with wild-type *mp* expression (*p* = 0.017). The relationship between resistance and the larval fat body phenotype in *multiplexin* mutants may suggest a role for the humoral immune response in this phenotype. Mutant flies may have altered antimicrobial peptide expression.

***Defective proboscis extension response 6.*** An intronic SNP (3L.10039434) in *Defective proboscis extension response 6* (*Dpr6*) was associated with variation in *P*. *rettgeri* load in flies reared on the low glucose diet (*p* = 9.54 x 10^-8^) and when data from flies reared on both diets were pooled (*p* = 3.51 x 10^-7^). *Dpr6* belongs to a family of genes thought to be involved in sensory perception of chemical stimulus, including gustatory perception of food, and contains an immunoglobulin domain that may be involved in cell-cell recognition [55]. Ubiquitous RNAi knockdown *of dpr6* resulted in a significant decrease in *P*. *rettgeri* load after infection (*p =* 0.0097).

**Fig 5:**
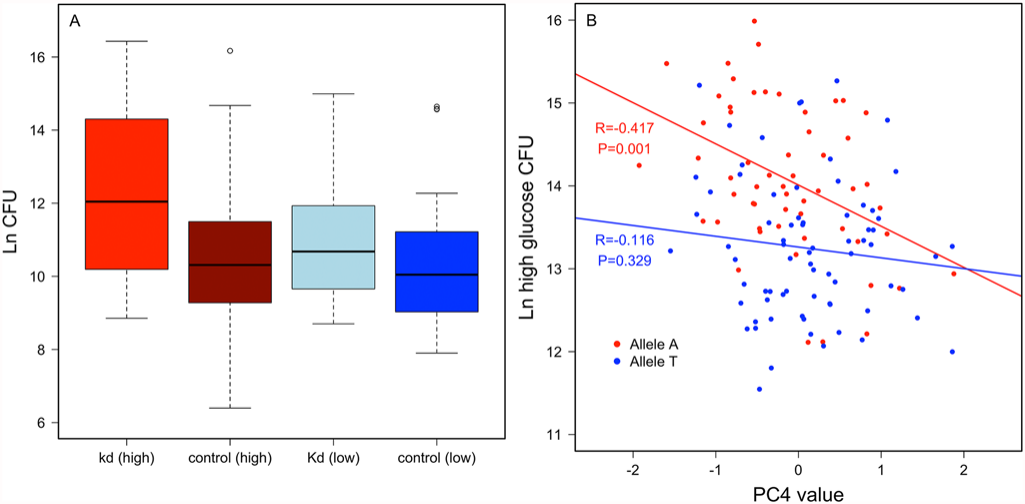
*Crinkled* has a diet-specific effect on immune defense. A.) validation experiment with RNAi knockdown (kd) shows natural log CFU 24 hours post infection with *P*. *rettgeri* compared to control (see S1 Table). B.) correlation between principal component 4 of nutritional indices on the high glucose diet and bacterial load on the high glucose diet polarized by allele in *crinkled* showing that this correlation between nutritional status and immune defense is driven by one allele.

***Crinkled.*** An intronic SNP (2L.15045678) in *crinkled* mapped for variable resistance specifically on the high glucose diet (*p* = 1.94 x 10^-7^), and less significantly when data from both diets were pooled (*p* = 4.29 x 10^-5^), but was not significantly associated with variation in *P*. *rettgeri* load when flies were reared on the low glucose diet (*p* = 0.0075). *Crinkled* encodes myosin VIIa, an actin-dependent ATPase. RNAi knockdown experiments for *crinkled* suggest that it does influence immunity in a diet-dependent manner. We used ubiquitous RNAi to knock down *ck* in flies reared on either the high glucose or low glucose diet. The knockdown had no significant effect on *P*. *rettgeri* load of flies reared on the low glucose diet (*p* = 0.45) but was marginally significant when flies were reared on the high glucose diet (*p* = 0.07; Fig. 5a). Further exploring the diet dependence, we found that Principle Component 4 of our nutritional indices measured on the high glucose diet correlated with *P*. *rettgeri* load in a *ck* allele-dependent manner. PC4 is strongly negatively correlated with bacterial load in flies homozygous for the A allele (*r* = -0.417, *P =* 0.0014) but is uncorrelated with load in flies bearing the T allele (*r = –* 0.115, *P =* 0.329; Fig. 5b). Since PC4 is loaded primarily with glucose, protein and glycogen, we also examined correlations between these NIs and *P*. *rettgeri* load within each *ck* allele in flies reared on the high glucose diet (S6 Fig). Mirroring the overall phenotypic data, free glucose levels trended toward negative correlation with *P*. *rettgeri* load in flies bearing the A allele (*r* = -0.23, *p* = 0.088) but not in flies bearing the T allele (*r* = -0.12, *p* = 0.32). Glycogen levels trended toward positive correlation with *P*. *rettgeri* load within the A allele (*r* = 0.20, *p* = 0.132) but not within the T allele (*r* = -0.07, *p* = 0.58).

***Sema-1a.*** An intronic SNP (2L.8630728) in *Sema-1a*, which encodes a semaphorin protein, fell just below our significance threshold on the high glucose diet (*p =* 1.42 x 10^-6^) and was much less significant on the low glucose diet (*p =* 0.0011). The dramatic difference in effect on the different diets suggested to us that *Sema-1a* might have diet-dependent effects on immunity. Semaphorins tend to be highly pleiotropic and play major roles in developmental processes [56]. RNAi knockdown of *Sema-1a* resulted in flies with marginally significantly higher *P*. *rettgeri* loads than controls on the low glucose diet (*p* = 0.081), on the high glucose diet (*p* = 0.088), and when the data from both diets were combined (*p* = 0.025).

***CG12869.*** A SNP (2R.10477114) 1594 bp upstream of functionally unannotated gene *CG12869* was significantly associated with *P*. *rettgeri* load in flies reared on the low glucose diet (*p* = 7.65 x 10^-7^) and approached significance in flies reared on the high glucose diet (*p* = 8.87 x 10^-5^) and when the data from both diets were combined (*p* = 1.49 x 10^-6^). While little is known about *CG12869*, the encoded protein is predicted to have carboxylesterase activity. RNAi knockdown of *CG12869* in flies reared on either the high-glucose or low-glucose diet resulted in modestly increased *P*. *rettgeri* loads when the data from both diets were combined (*p*_*pooled*_ *=* 0.047), although not when either diet is considered independently (low glucose: *p* = 0.133, high glucose: *p* = 0.164).

***G protein-coupled receptor kinase 2.*** *Gprk2* was previously associated with defense response to bacteria through interaction with *cactus* and is required for normal AMP production [57]. It is also involved with several biological processes that might be influenced by nutritional environment including hedgehog signaling and regulation of appetite {Cheng:2012kd, Chatterjee:2010df}. An intronic SNP in *Gprk2* (3R.27273757) yielded a significant SNP-by-diet interaction predicting *P*. *rettgeri* load (p = 7.75 x 10^-8^). RNAi knockdown of *Gprk2* resulted in increased *P*. *rettgeri* load relative to control flies (*p* = 0.016), consistent with the results of Valanne *et al*. [57], who found that *Gprk2* disruption reduced resistance to infection by *Enterococcus faecalis*. We found no distinction between knockdown on high-glucose versus low-glucose diets (*p*_knockdown_ = 0.08, *p*_diet_ = 0.04, *p*_interaction_ = 0.94).

**Thioester-containing protein 2.** Thioester-containing proteins (TEPs) are opsonins that promote phagocytosis and parasite killing in invertebrates, including phagocytosis of Gram-negative bacteria [58]. TEPs are homologous to vertebrate complement C3 and macroglobulins, and *Drosophila TepII* has previously been shown to evolve under adaptive positive selection in the presumptive pathogen-binding domain [59]. *P*. *rettgeri* load was determined by a significant diet*SNP interaction for four nonsynonymous SNPs in *TepII* (*p* = 5.97 x 10^-7^) and an additional synonymous SNP in tight disequilibrium (*p* = 7.00 x 10^-7^). RNAi knockdown of *TepII* resulted in reduced immune defense (*p* = 0.0017), independent of diet (*p* = 0.94), which is consistent with the known role of *TepII* in insect immunity.

***Similar.*** An intronic SNP in *similar* (3R.25909307) showed a significant Diet*SNP interaction (*p* = 9.17 x 10^-7^). *Sima* is involved in protein dimerization and signal transduction and has been associated with response to stress. RNAi knockdown of *similar* resulted in increased *P*. *rettgeri* load after infection of flies reared on the low glucose diet (*p =* 0.005) but not on the high glucose diet (*p =* 0.28). Variants of *similar* may influence how an individual responds to a nutrient-poor diet which in turn may influence their ability to resist infection.

***Kinesin heavy chain 73 and Src64B.*** A synonymous SNP and a SNP in the 3′ UTR of *Khc-73* were associated with variation in bacterial load when data from both diets are pooled (3.1 x 10^-7^ and 5.48 x 10^-8^, respectively), and an intronic SNP (3L.4603286) in *Src64b* mapped highly significantly on each diet (low glucose: *p* = 1.41 x 10^-7^; high glucose: *p* = 2.50 x 10^-7^) and when the data from both diets were pooled (*p* = 3.34 x 10^-9^). *Khc-73* is a microtubule motor protein [53] and *Src64b* is a tyrosine kinase with a wide range of reported phenotypes including cellular immune response [60]. RNAi knockdown of either gene did not result in any significant change in systemic *P*. *rettgeri* load after infection (*Khc-73*: *p* = 0.67, *Src64b*: *p =* 0.97). Thus, neither of these mapped genes validated by our RNAi knockdown criteria. This could be because the two genes are false positive map results or because the RNAi failed to adequately block protein synthesis in the knockdown experiment.

**Other candidate genes.** We mapped SNPs associated with variation in post-infection *P*. *rettgeri* load in the genes *elk*, *fruitless*, *tout velu*, *CG42524*, *CG7991*, *CG4835* and *CG15544* (Table 5), but we were unable to establish RNAi knockdowns for these and were thus unable to test whether disruption of these genes influences resistance to infection.

**Nearest-neighbor negative controls.** It is unknown what proportion of genes in the genome could conceivably yield immune defense phenotypes when ubiquitous RNAi disrupts their expression. To estimate the false-positive rate on our RNAi knockdowns, we additionally knocked down several arbitrary genes that are physically adjacent to our mapped genes but are not known to have any immune function. Whereas 9 out of 13 of our mapped candidate genes yielded defense phenotypes upon RNAi knockdown, only one out of the five arbitrary neighboring genes yielded an immunity phenotype. Little is known about the function of that arbitrary gene whose knockdown resulted in a modest decrease in *P*. *rettgeri* load (*p* = 0.029) (*CG34356*), but it has been shown to be involved in protein phosphorylation [61]. Our rate of 9 in 11 positive knockdown experiments among the mapped candidates is a significant excess over the 1 in 5 negative control genes that gave immune phenotypes (Fisher’s Exact Test: *p* = 0.018), giving us confidence that the majority of our mapped genes are true positive results.

### Genome-wide association mapping and NI correlations with *Diptericin* genotype as a covariate

*Diptericin* is a classical immunity gene with a large effect in our study. We reasoned that genotype at *Diptericin* might mask genes with smaller effects, and that we could increase power to detect diet-dependent variants by controlling for *Diptericin* genotype. Furthermore, there was significant linkage disequilibrium between SNPs in *Diptericin* and other mapped SNPs (S7 Fig). We therefore re-conducted the genome-wide association analysis with the addition of *Diptericin* genotype as a covariate that could take on three possible states: the arginine versus serine variants at position 2R.14753589 and the premature stop codon (although the two DGRP lines carrying the premature stop codon also carried the serine variant, we classified them separately because they were phenotypically so extreme). Lines carrying residual heterozygosity at *Dpt* were treated as having missing data for the *Dpt* genotype. All GWAS results and knockdown experiments reported to this point were mapped *without Dpt* genotype as a covariate. Unexpectedly, instead of revealing new genes that predict immune phenotype, inclusion of *Dpt* genotype in the mapping model caused the number of significant SNPs (*p* < 10^-6^) to drop from 19 to only 4 when the data from both diets were pooled, from 11 to 2 on the low glucose diet only, and from 7 to 1 on the high glucose diet only. Inclusion of *Dpt* as a covariate greatly improved the observed fit of our q-q plots to the null expectation, eliminating experiment-wide *p*-value inflation (S4 Fig). We observed an increase in the number of SNP-by-diet interactions from 77 to 88 when *Dpt* genotype is included as a covariate (S4 Fig, S3 Table). Only one SNP (2L.13072327; located in a small RNA) was significant in both the original models and when *Dpt* genotype was used as a covariate. For the SNPs significant for the interaction effect, 66 were significant in both methods, 14 were specific to mapping without *Dpt* as a covariate, and 25 were specific to mapping with *Dpt* as a covariate. As shown in S4 Fig, there is generally good agreement between the two methods for interaction, although both are quite inflated.

To assess whether mapping with *Diptericin* genotype as a covariate provided reliable results, we performed the same RNAi knockdown experiments as described above with two new candidates genes. Both resulted in increased pathogen load when knocked down (S5 Fig, S1a Table). Briefly, these genes were CG33090, a beta-glucosidase, and CG6495, a gene of unknown function that was significantly induced upon infection in a previous study [62]. We additionally chose to validate CG12004, which mapped with a P-value that missed our significance threshold (*p* = 4.72 x 10^-6^), but that has been previously shown to be involved in defense response to fungus [63]. Knockdown of CG12004 resulted in a marginally significant increase in pathogen load (P = 0.0514).

We reexamined the correlations between nutritional indices and bacterial load when *Dpt* variant was included as a covariate in the regression. In all cases, the model with *Dpt* variant was a better fit than the model that did not include *Dpt* genotype (S4 Table). On the high glucose diet, the correlation between free glucose level and bacterial load becomes slightly less significant (*p =* 0.078 vs. *p =* 0.033 previously), while the correlation between soluble protein and bacterial load became more significant (*p =* 0.036 vs. *p =* 0.061 previously). No principal components on the low glucose diet became significant with *Dpt* as a covariate. However, on the high glucose diet, PC3 became marginally significant (*p =* 0.052 vs. 0.194 without considering *Dpt* genotype) and PC4 remained significant (*p =* 0.007 vs. 0.001 without considering *Dpt* genotype).

## Discussion

We found the *Drosophila* Genetic Reference Panel to be highly variable for resistance to *P*. *rettgeri* infection. We also determined that the severity of bacterial infection increased dramatically when flies were reared on a high-glucose diet, and the flies became hyperglycemic and hyperlipidemic. Relative quality of immune defense was highly correlated across the two diets, indicating strong genetic determination of the defense phenotype. However, we also observed a significant genotype-by-diet interaction shaping defense. Specifically, there were several lines that suffered disproportionately severe infections after rearing on the high-glucose diet, although these lines fell closer to the center of the resistance distribution when they were reared on the low-glucose diet. We did not find any lines that showed markedly higher resistance on the high-glucose diet. We were able to identify several genes that contributed to variation in resistance on both diets.

Not only did severity of infection increase with elevated dietary glucose, the flies became hyperglycemic, hyperlipidemic, and had elevated glycogen stores after rearing on the high-glucose diet. Because both glucose levels and infection severity increased with rearing on the high glucose diet, we predicted that those two traits would also be genetically correlated. Unexpectedly, however, free glucose levels were *negatively* correlated with severity of infection across genotypes when flies were reared on the high glucose diet (the two traits were uncorrelated on the low glucose diet). We observed an even stronger correlation between resistance and a principal component that was positively loaded with free glucose and negatively loaded with glycogen stores. Because metabolic measurements were taken from uninfected flies, they indicate genetic capacity to assimilate or manage the excess dietary glucose in the absence of the pathogen. The genetic correlation with infection severity indicates that resistance to *P*. *rettgeri* infection is linked in some way to glucose metabolism, uptake, and/or conversion to and from glycogen. Our map results suggest that this effect is partially mediated by the *crinkled* gene, which encodes a myosin VIIa cytoskeletal ATPase. We identified a polymorphism in *crinkled* that highly significantly predicted bacterial load when flies were reared on the high glucose diet, although not on the low glucose diet. Flies bearing the rarer allele show strongly negatively correlated glucose levels and pathogen loads. Furthermore, independently determined expression of the *crinkled* gene [64] correlates with resistance to *P*. *rettgeri* and our observed glucose level. Full characterization of the mechanism by which *crinkled* shapes immunity and glucose metabolism will require future study.

We were more generally able to map several genes that contribute to phenotypic variation in immune performance, both in diet-specific and diet-independent manners. The mapped polymorphisms were highly significantly enriched for being nonsynonymous and for lying within or very near genes. RNAi knockdown confirmed roles for the mapped genes in resistance to *P*. *rettgeri*, with 82% of the knockdowns of mapped genes resulting in altered pathogen loads. In contrast, we found defense phenotypes after knockdown of only 17% of negative control genes that are chromosomally linked to mapped genes but were otherwise arbitrary.

Only a small handful of the mapped genes had annotated immune function. Instead, we identified genes encoding proteins annotated in processes such as feeding behavior and cytoskeletal trafficking. This is a fully expected outcome of the experiment, and such genes are precisely what GWAS studies are designed to detect. Functional variation in dedicated immune genes is probably subject to strong natural selection in the wild, and most variation is probably quickly purged from the population. In contrast, however, populations may retain genetic variation that results in smaller effects on resistance, especially when the primary selection on the gene is for a function other than immune defense. Such genetic variants can then cause a large proportion of the observed phenotypic variance in natural populations, and in mapping panels derived from natural populations, such as the DGRP. The effect on immune defense of knocking down the mapped genes by RNAi was small relative to what might be expected from disruption of core components of the immune system. For this reason, it is unsurprising that these genes have not been discovered in previous mutation screens for susceptibility to bacterial infection. That we are able to map and confirm many of these non-conventional genes opens the possibility of whole new avenues of research and illustrates the value of unbiased genome-wide mapping relative to candidate gene based studies. This result also suggests that resistance to infection, especially in the context of dietary variation, is best viewed as a synthetic trait of the whole organism phenotype and is not determined solely by the canonical immune system. Genes that influence any number of developmental or metabolic processes may carry variation that directly or indirectly influences the ability of the organism to resist infection.

At the outset of this experiment, we might have hypothesized that the genetic basis for immunological sensitivity to diet would map to stereotypical metabolic processes, either because of crosstalk between metabolic and immune signaling, varied ability to incorporate metabolites during development, or variation in the capacity to sequester nutrients from pathogens. However, our mapping did not uncover the most obvious potential metabolic processes, such as insulin-like signaling, carbohydrate metabolism, or energetic storage. Instead, we identified genes with highly diverse function, which indicates a much more nuanced and complex interaction between dietary intake and immune defense. Importantly, because the flies in our study were reared from egg-to-adult on the experimental diet of interest, we do not distinguish between defense-impacting effects that arise during development versus those that manifest during the response to infection. It is important to bear in mind that the effects of allelic variation in the mapped genes could manifest at any stage of development or in any aspect of host physiology that may ultimately influence antibacterial defense. Determining the mechanisms by which the mapped genes influence resistance will require considerable additional study. In most cases, the RNAi knockdowns of mapped genes confirmed an effect of immune phenotype, but did not necessarily recapitulate diet-specific effects on resistance. While RNAi knockdown is a useful tool for confirming the role of mapped genes in immune defense, it is expected that the effect of RNAi knockdown will be much larger than the difference in phenotype between two alleles of the gene. Thus, where the SNP variants may cause modest modification of defense phenotype—perhaps revealed only under certain dietary environments—the RNAi knockdowns are more of a sledgehammer whose effects will be seen under all dietary conditions.

One of the variants that most significantly predicted pathogen load irrespective of diet was an amino acid polymorphism in the canonical antibacterial peptide Diptericin. This was surprising to us, as previous candidate gene studies had failed to detect major effect of allelic variation in *Diptericin* or any other antimicrobial peptide gene on resistance to *Serratia marcescens*, *Enterococcus faecalis*, *Lactococcus lactis*, or *Providencia burhodogranariea* [26–28]. Our interpretation had been that AMPs are plentiful and functionally redundant [52], such that minor variation in any one peptide would not have major effect on organism-level resistance. However, in followup experiments we have confirmed that the Serine/Arginine variant mapped in the present study is a strong predictor of resistance to some but not all Gram-negative bacterial pathogens (Unckless *et al* in prep). Thus, it would appear that the relative importance of Diptericin, and by extension presumably other antibacterial peptides, depends on the agent of infection. Moreover, we have found an independent mutation in natural populations of *Drosophila simulans* that converges on a Serine/Arginine polymorphism at the same *Diptericin* codon, with the same consequence for relative resistance to this suite of bacteria. Surprisingly, natural populations of both *D*. *melanogaster* and *D*. *simulans* are additionally polymorphic for apparent loss-of-function mutations at *Diptericin*, and flies carrying these variants are highly susceptible to infection by *P*. *rettgeri* and other bacteria [38](Unckless et al in prep). The collective data indicate a complex evolutionary history of *Diptericin* that includes convergent evolution of selectively balanced polymorphisms in two species, with variation in relative resistance to a subset of pathogens.

We found that infection by the endosymbiont *Wolbachia pipientis* is associated with modest but significant resistance to infection by *P*. *rettgeri*. Previous studies have not found differences in *Wolbachia*-infected vs. uninfected flies in immune system activity or resistance to infection by secondary bacteria, including *P*. *rettgeri* [42,43,65]. Our present study is substantially larger than these others, and therefore may have greater power to detect small protective effects of *Wolbachia* infection. Unlike previous studies which have compared *Wolbachia-*infected flies to genetically matched lines which were cured of *Wolbachia* using antibiotics, our present study cannot fully distinguish between the effects of *Wolbachia* and host genotype. For example, *Wolbachia* infection status could be associated with general health of the lines and therefore resistance to *P*. *rettgeri* infection, or *Wolbachia* infection could be significantly associated with a genetic polymorphism that also predicts resistance to *P*. *rettgeri*. Presence of *Wolbachia* was weakly associated with a decrease in soluble protein in the present study (*p* = 0.046), and has been previously shown to alter fly physiology by buffering the effects of excess or deficit in dietary iron [66] and by modulating other metabolic processes including insulin signaling [67]. These physiological impacts may suggest indirect mechanisms by which *Wolbachia* infection could confer weak protection against infection by pathogens like *P*. *rettgeri*.

In summary, we have shown that natural genetic variation for immune defense can be attributed to variation in several genes, with both diet-dependent and diet-independent effects. We also find that metabolic indices are correlated with immune defense when flies are reared on a high glucose diet. Importantly, several of the mapped genes would not be considered conventional “immune” genes, yet we confirm with RNAi knockdown that they pleiotropically contribute to immune defense. The genes mapped in this study harbor allelic variation that shapes the quality of immune defense, and thus may be instrumental in the evolution of resistance to bacterial infection in natural populations experiencing varied dietary environments.

## Materials and Methods

### *Drosophila* and bacterial strains used

The *Drosophila* Genetic Reference Panel (DGRP; [39]) is a collection of 192 lines that have been inbred to homozygosity and whose complete genomes have been sequenced. Each line is derived from an independent wild female captured in a fruit market in Raleigh, NC, USA in 2003. We used 172 of the most robust lines for this study, though the exact number and composition of lines varied slightly among replicate blocks of the experiment.

Bacterial infections were performed using *Providencia rettgeri* strain Dmel, which was isolated as an infection of a wild-caught *D*. *melanogaster* [68]. *P*. *rettgeri* are Gram-negative bacteria in the family Enterobacteriaceae, and are commonly found in association with insects and other animals. Injection of the Dmel strain of *P*. *rettgeri* into *D*. *melanogaster* under the conditions used here results in a highly reproducible initial dose of bacteria that proliferates 100–1000 fold over the first 24 hours post-infection, depending on the host fly genotype, with low to moderate host mortality [51]. Bacterial load at 24 hours post-infection correlates strongly with risk of host mortality [38], but pathogen load as a phenotype does not confound resistance and tolerance mechanisms in the way that survivorship does.

### *Drosophila* diets

We used two experimental diets that varied in glucose content but otherwise had the same composition. The base diet was composed of 5% weight per volume Brewer’s yeast (MP Biomedicals, Santa Ana, CA) and 1% *Drosophila* agar (Genesee Scientific, San Diego, CA). The high-glucose diet contained 10% glucose (Sigma-Aldrich Corp., St. Louis, MO) while the low-glucose diet contained 2.5% glucose. All diets were supplemented with 800 mg/L methyl paraben (Sigma-Aldrich Corp., St. Louis, MO) and 6 mg/L carbendazim (Sigma-Aldrich Corp., St. Louis, MO) to inhibit microbial growth in the food. RNAi knockdown experiments for SNPs significant when data from both diets were pooled were performed on the “standard diet” which contained 8.2% glucose and 8.2% Brewer’s yeast. Each DGRP line was split and raised in parallel on both diets for at least three generations prior to the start of the experiment to control parental and grandparental effects within dietary treatments, and experimental flies were reared egg-to-adult in the dietary condition being assayed. We recognize that our diets differ in total caloric content as well as protein to carbohydrate ratios [4,13,14]. It is possible that *Drosophila* change their feeding behavior on the two diets, and that there may even be genetic variation for feeding behavioral response to diet. Our goal in this study is to determine the consequences of excess dietary glucose while remaining agnostic as to the precise cause of any altered nutritional assimilation.

### Method of bacterial infection

*Providencia rettgeri* strain Dmel was grown overnight to stationary phase in Luria-Bertani (LB) broth at 37°C prior to each infection day. On the morning of infections, stationary cultures were diluted in sterile LB broth to A_600_ = 1.0. Male flies from each DGRP line were infected in the lateral scutum of the thorax by pricking with needles (0.10mm, Austerlitz Insect Pins, Prague, CR) that had been dipped in the diluted bacterial suspension, delivering approximately 1000 bacteria to each infected fly. Infections were performed in three blocks for each diet with each block containing all or nearly all DGRP lines under study. Each block for each diet was performed on a different day, with replicate blocks for the two diets interspersed on alternating weeks. Three researchers performed the infections on each experimental day, with lines assigned randomly to infectors within each block. Males aged 3–6 days post-eclosion were infected from each line. All flies were maintained in an incubator at 24°C on a 12-hour light/dark cycle. Infections were delivered approximately 2–4 hours after “dawn” from the perspective of the flies.

Approximately 24 hours after infection, males were homogenized in groups of 3 in 500 ul sterile LB broth. The homogenate was plated on standard LB agar plates using a robotic spiral plater (Don Whitley Scientific). Plates were incubated overnight at 37°C, and the resultant colonies were counted using the ProtoCOL system associated with the plater. *P*. *rettgeri* grows readily on Luria agar at 37°C, but the endogenous microbiota of *D*. *melanogaster* does not. Thus, we were able to capture colonies derived from viable infecting *P*. *rettgeri* without interference from the *Drosophila* gut microbiota. Counted colonies were visually inspected for morphology consistent with *P*. *rettgeri*, and homogenates from sham-infected flies always failed to yield bacterial colonies within the assay period. We used systemic pathogen load at 24 hours post-infection as our measure of immune defense.

In total, 6–9 data points representing 18–27 flies were collected from each line on each diet (high and low glucose). The total experiment consists of 1429 data points representing 4287 flies on the low glucose diet, and 1396 data points representing 4188 flies on the high glucose diet.

### Nutritional indices in the DGRP

We queried a series of nutritional indices in flies reared on each diet. Each metabolite was assayed in three replicates on flies reared on each diet. Males were aged 3–6 days post-eclosion, then 10 live males were weighed using a MX5 microbalance (Mettler-Toledo, Columbus, OG) and homogenized in 200 μL buffer (10 mM Tris, 1 mM EDTA, pH 8.0 with 0.1% v/v Triton-X-100) using lysing matrix D (MP Biomedicals, Santa Ana, CA) on a FastPrep-24 homogenizer (MP Biomedicals, Santa Ana, CA). An aliquot of 50 microliters were frozen immediately while 150 microliters were incubated at 72 degrees C for 20 minutes to denature host proteins. Nutrient assays were performed with minor modifications of the procedures described in [69] using the following assay kits from Sigma-Aldrich (St. Louis, MO): glucose with the oxidase kit (GAGO-20); glycogen using the glucose kit and amyloglucosidase from *Aspergillus niger* (A7420) in 10 mM acetate buffer at pH 4.6; free glycerol and triglycerides using reagent kits F6428 and T2449, respectively. Soluble protein was assayed with the DC Protein Assay (BIO-RAD, Hercules, CA). Each metabolite was assayed on each pool of weighed and homogenized flies.

### Data analysis

Mixed effect linear models were used to test for genetic and other contributions to phenotypic variation in systemic pathogen load and nutritional indices. Overall genetic main effects on systemic pathogen load were tested with the model

$$Y_{ijklmno}=\;\mu +\;Wolb_{i}+\;Diet_{j}+\;Line_{k}(Wolb_{i})\;+\;Infector_{l}+\;Plater_{m}+\;Block_{n}(Diet_{o})\;+\;Diet_{o}*Line_{k}(Wolb_{i})\;+\;\varepsilon_{ijklmno}$$

where Y is the natural log-transformed measure of pathogen load for each data point, Wolb_*i*_ (*i* = 1,2) has a fixed effect and indicates whether the line is infected with the endosymbiotic bacterium *Wolbachia pipientis*, Diet_*j*_ (*j* = 1,2) has a fixed effect and indicates which of the two diets the flies were reared on, Infector_*l*_ (*l* = 1,3) has a fixed effect and is used to test whether the experimentor performing the infections influenced ultimate pathogen load, and Plater_*m*_ (*m* = 1,2) has a fixed effect and indicates which of two spiral platers were used to plate the sample. Block_*n*_(Diet_*o*_) (*n* = 1,3) has a fixed effect nested within the effect of Diet, and is used to test for differentiation among the three replicate blocks for each dietary treatment. Line_*k*_(Wolb_*i*_) (*k* = 1,172) is assumed to have a random effect, and is used to test the influence of genetic line on pathogen load within *Wolbachia*-infected and *Wolbachia*-uninfected classes. The interaction Diet_o_*Line_*k*_(Wolb_*i*_) is considered to have a fixed effect and tests whether genetic lines differ in their responsiveness to the two diets. This model was run in SAS 9.3 (Cary, NC) using the “mixed” procedure.

We determined line means for each nutritional index using abundance of metabolite per fly. The model used was analogous to that used for bacterial load:

$$Y_{ijklmno}=\;\mu +\;Wolb_{i}+\;Diet_{j}+\;Line_{k}(Wolb_{i})\;+\;Block_{n}(Diet_{o})\;+\;Diet_{o}*Line_{k}(Wolb_{i})\;+\;\varepsilon_{ijklmno}$$

Again, all factors were considered to be fixed except Line(Wolb) and Diet*Line(Wolb) and best linear unbiased predictors (BLUPs) were extracted for further analysis. For comparisons between diets, the model used was Nutrient/fly~Wolb+Line(Wolb)+Block.

To determine whether there was a genetic signature of a “metabolic syndrome” that may influence immune defense, we performed principal component analysis using the BLUPs extracted for each nutritional index. This analysis was implemented in R with the *prcomp* function with *tol* = 0.1 and unit variance scaling on. The principal component values for were then tested for correlation with bacterial load. This analysis was done for each diet individually.

### Genome-wide association mapping

The set of SNPs for mapping was described in Huang *et al*. (in revision) and consists of only SNPs with minor alleles present in at least four of the lines (MAF >2%; 2415518 total SNPs). For bacterial load (Ln CFU), we used SAS to run the following model:

LnCFU = m+SNP_*i*_+Diet_*j*_+SNP_i_*Diet_*j*_+Block_*k*_(Diet_*j*_)+Wolb_*l*_+Infector_*m*_+Plater_*n*_+Line_*o*_(SNP_*i*_)+e_*ijklmno*_, where all factors were fixed except Line(SNP). P-values for the main effect of SNP and the SNP*Diet interaction were obtained for each SNP. We also ran the model separately on data obtained from flies reared on each of the two diets to obtain significance values for each SNP on each diet independently. These models were LnCFU = m*+*SNP_*i*_+Block_*j*_+Wolb_*k*_+Infector_*l*_+Plater_*m*_+Line_*n*_(SNP_*i*_)+e_*ijklmn*_. We considered SNPs that mapped with significance level of *p* < 10^-6^ to be nominal positive hits and candidates for RNAi knockdown experiments. This *p-*value corresponds to a false discovery rate of 5–10% depending on the precise analysis being performed.

### GO term analysis

To correct for gene size, we used GOWINDA [47] to test for the enrichment of particular functional groups. Here we relax our significance threshold to include all SNPs with *p*<10^-5^. This allows for more power through the inclusion of additional SNPs. Relaxing the P*-*value threshold even further had little effect on GO enrichment results. Significantly associated SNPs for each treatment (low glucose, high glucose, main effect) were used with a background SNP set consisting of all SNPs used in the GWAS. GO slim [48] terms were used to reduce redundancy in GO categories. GOWINDA was run using *gene* mode, including all SNPs within 1000bp of a gene, a minimum gene number of 5, and with 100,000 simulations. We report all GO terms with a nominal P-value less than 0.1.

### RNAi knockdown experiments for genes containing SNPs significantly associated for bacterial load

For all SNPs with P-values meeting our significance threshold and falling within 1000 bp of an annotated gene, we performed the infection assay described above on RNAi knockdown lines from the Vienna Drosophila RNAi Center (Vienna, Austria), if available.

To test the effect of the gene on resistance to infection, we crossed each RNAi line to a line carrying the ubiquitous driver (Act5C-Gal4/Cyo or da-Gal4) and infected F1 offspring of the knockdown genotype. We compared the immune defense in these F1 offspring to that of F1 progeny from the driver line crossed to the background genetic line of the RNAi transformant. Unless otherwise indicated, we performed RNAi knockdown experiments using a standard diet (1:1 glucose to yeast ratio, but more calorie dense than our high and low glucose diets—see methods). For those SNPs that showed a diet-specific effects, we performed RNAi knockdown experiments on the experimental high and low glucose diets.

It is completely unknown what proportion of genes throughout the genome might yield an immune phenotype when expression is repressed. To test whether genes containing our significantly associated SNPs were more likely to have an immune phenotype than a set of arbitrary genes from the genome, we also performed RNAi knockdown experiments on the genes that were physically close to those of interest but not known to be involved in immunity and not essential for viability. We refer to these as “nearest neighbor controls”.

## Supporting Information

S1 Table A) RNAi knockdown experiments on standard diet and B) RNAi knockdown experiments on high and low glucose diets. (DOCX)

S2 Table GO enrichment results. (XLSX)

S3 Table Significantly associated SNPs from genome-wide association study controlling for *Dpt* genotype. (DOCX)

S4 Table Model comparisons for correlations between immune response and nutritional phenotypes when *Diptericin* state is ignored or accounted for. (DOCX)

S1 Fig Sorted boxplots for pathogen load 24-hours post infection (Ln CFU) for each DGRP line on a) high glucose diet and b) low glucose diet. (TIFF)

S2 Fig Comparison of *P*. *rettgeri* load (Ln CFU) 24 hours post infection in lines infected and uninfected with *Wolbachia*, showing *Wolbachia* is associated with a lower pathogen load. Bacterial load data is pooled across both diets.
(TIFF)

S3 Fig Manhattan plot from genome-wide associations a) values from both diets pooled, b) interaction, c) high glucose diet, d) low glucose diet. Dotted line represents nominal *p*-value cutoff of 10^-6^.
(TIFF)

S4 Fig Quantile-Quantile plots from genome-wide associations a) values from both diets pooled, b) interaction, c) high glucose diet, d) low glucose diet. Dotted line is one to one. Black dots from standard mapping approach; red dots from mapping when *Diptericin* state is used as a covariate.
(TIFF)

S5 Fig RNAi knockdown experiments with mapped genes a) mean difference between knockdown (kd) and control is plotted for SNPs that did not have a diet-specific effect; values above zero indicate knocked down flies have higher loads than controls (*p*<0.001***, *p*<0.01**, *p*<0.05*), b) validations for genes with putative diet-specific effects. *Pooled* refers to genes containing SNPs that mapped when data from both diets were pooled, *interaction* refers to genes with SNPs that mapped for the interaction term, *Dpt covariate* refers to genes containing SNPs that mapped when *Dpt* allele was included as a covariate and *nearest neighbor* refers to genes selected as position matched controls.
(TIFF)

S6 Fig Correlation between two main components of principal component 4 a) glucose and b) glycogen per fly and Ln CFU on the high glucose diet, polarized by allele in *crinkled*. (TIFF)

S7 Fig Pairwise linkage disequilibrium (*D’*) between significant SNPs in genome-wide associations when data from both diets were pooled. Only one SNP per gene was used to reduce signal from physical linkage. (*p*<0.0001***, *p*<0.001**, *p*<0.05*)
(TIFF)
