# Supplementary material for: The Complex Contributions of Genetics and Nutrition to Immunity in Drosophila melanogaster
Source: PLoS Genet. 2015 Mar 12;11(3):e1005030. doi: 10.1371/journal.pgen.1005030 (PMC4357385; doi:10.1371/journal.pgen.1005030)
Supplement: S1 Table — (DOCX) [file pgen.1005030.s001.docx]

**Table S1a.** RNAi knockdown experiments on standard diet

| **symbol** | **CG** | **Mapped** | **VDRC ID** | **Collection** | **Driver** | **KD effect (SE)** | **P-value** |
| --- | --- | --- | --- | --- | --- | --- | --- |
| *CG15544* | *CG15544* | pooled | 39997 | GD | Actin5C | 0.084 (0.702) | 0.9018 |
| *dpr6* | *CG14162* | pooled | 41161 | GD | Actin5C | 1.338 (0.532) | **0.0097** |
| *Khc-73* | *CG8183* | pooled | 105984 | KK | Actin5C | 0.388 (0.117) | 0.6679 |
| *mp* | *CG42543* | pooled | 100846 | KK | Actin5C | -1.338 (0.552) | **0.0284** |
| *Src64B* | *CG7524* | pooled | 35252 | GD | Daughterless | 0.019 (0.153) | 0.9716 |
| *CG7991* | *CG7991* | pooled | 35309 | GD | Actin5C | 0.344 (0.269) | 0.6461 |
| *Grpk2* | *CG42543* | interaction | 101463 | KK | Actin5C | -1.764 (0.737) | **0.0165** |
| *sima* | *CG45051* | interaction | 106187 | KK | Actin5C | -2.163 (0.765) | **0.0005** |
| *TepII* | *CG7052* | interaction | 106997 | KK | Actin5C | -1.841 (0.671) | **0.0016** |
| *CG12004* | *CG12004* | Dpt cov | 101732 | KK | Actin5C | 1.561 (0.335) | *0.0514* |
| *CG33090* | *CG33090* | Dpt cov | 28033 | GD | Actin5C | 2.92 (0.438) | **0.0178** |
| *CG6495* | *CG6495* | Dpt cov | 42794 | GD | Actin5C | 5.118 (0.762) | **1.00E-04** |
| *CG7025* | *CG7025* | NN | 49706 | GD | Actin5C | -0.471 (0.418) | 0.4679 |
| *CG8641* | *CG8641* | NN | 110183 | KK | Actin5C | -0.09 (0.345) | 0.9015 |
| *CG18682* | *CG18682* | NN | 101589 | KK | Actin5C | -0.466 (0.651) | 0.4696 |
| *CG34356* | *CG34356* | NN | 109790 | KK | Actin5C | -1.872 (0.214) | **0.0293** |
| *Mapmodulin* | *CG5784* | NN | 100283 | KK | Actin5C | -0.994 (0.417) | 0.2602 |
|  |  |  |  |  |  |  |  |

Bold indicates significant (P<0.05); Italics indicate (0.05<P<0.1); SNPs mapped on pooled=data from both diets pooled, interaction=interaction term of mapping, Dpt cov=mapped when Dpt variant was used as a covariate, nearest neighbors to mapped genes

**Table S1b.** RNAi knockdown experiments on high and low glucose diets

| **Symbol** | **CG** | **Mapped on** | **VDRC ID** | **Coll** | **Driver** | **High (cont)** | **High (KD)** | **Low (cont)** | **Low (KD)** | **P(KD)** | **P(diet)** | **P(int)** | **P(High)** | **P(Low)** |
| --- | --- | --- | --- | --- | --- | --- | --- | --- | --- | --- | --- | --- | --- | --- |
| *TepII* | *CG7052* | Interaction | 106997 | KK | Actin5C | 12.07 | 11.93 | 10.31 | 10.19 | 0.617 | **0.004** | 0.945 | 0.864 | 0.88 |
| *sima* | *CG45051* | Interaction | 106187 | KK | Actin5C | 12.14 | 13.83 | 10.06 | 13.59 | **0.026** | 0.111 | 0.366 | 0.28 | **0.005** |
| *Gprk2* | *CG42543* | Interaction | 101463 | KK | Actin5C | 12.14 | 11.83 | 10.06 | 11.48 | 0.721 | 0.132 | 0.269 | 0.79 | 0.136 |
| *ck* | *CG7595* | High glucose | 100010 | KK | da | 10.33 | 11.86 | 10.10 | 10.61 | **0.049** | 0.174 | 0.343 | *0.073* | 0.447 |
| *Sema-1a** | *CG18405* | High glucose | 104505 | KK | da | 11.79 | 12.91 | 9.97 | 11.22 | **0.025** | **<0.001** | 0.827 | *0.088* | *0.081* |
| *CG12869* | *CG12869* | Low glucose | 100474 | KK | da | 10.34 | 11.70 | 10.10 | 11.15 | **0.047** | 0.512 | 0.72 | 0.164 | 0.133 |

Bold indicates significant (P<0.05); Italics indicate (0.05<P<0.1)
