# Supplementary material for: The Complex Contributions of Genetics and Nutrition to Immunity in Drosophila melanogaster
Source: PLoS Genet. 2015 Mar 12;11(3):e1005030. doi: 10.1371/journal.pgen.1005030 (PMC4357385; doi:10.1371/journal.pgen.1005030)
Supplement: S3 Table — (DOCX) [file pgen.1005030.s003.docx]

**Table S3.** Significantly associated SNPs from genome-wide association study controlling for *Dpt* genotype

| **SNP** | **gene** | **class** | **A1** | **A2** | **MAF** | ***p_high_*** | ***p_low_*** | ***p_pooled_*** |
| --- | --- | --- | --- | --- | --- | --- | --- | --- |
| 2L.10764719 | *CG6495* | syn. | C | A | 0.10 | 3.04E-04 | 3.00E-06 | 2.21E-06 |
| 2L.13072327 | *s2_48__2_1156898* | snRNA | T | G | 0.45 | 4.18E-06 | 2.34E-05 | 3.22E-07 |
| 2L.14500350 | *tRNA:P:35Bd* | dn(4401) | C | A | 0.19 | 1.65E-06 | 0.0040 | 7.12E-05 |
| 2L.14343666 | *CG33090* | syn. | C | G | 0.39 | 4.91E-06 | 1.26E-05 | 2.78E-07 |
| 2L.14355395 | *Rab14* | 5’UTR | C | G | 0.05 | 0.0233 | 1.03E-06 | 5.09E-05 |
| 2L.19183703 | *gammaTub37C* | dn(160) | T | G | 0.22 | 1.15E-06 | 0.0012 | 1.90E-05 |
| 2R.6777267 | *CG30020* | syn. | A | G | 0.48 | 2.36E-04 | 4.13E-06 | 2.36E-06 |
| 2R.12699220 | *CG5550* | up(4876) | C | A | 0.03 | 0.0277 | 2.82E-06 | 3.24E-05 |
| 2R.12699239 | *CG5550* | up(4857) | C | T | 0.03 | 0.0277 | 2.82E-06 | 3.24E-05 |
| 3L.2399650 | *CG13800/CG42669* | intron | C | T | 0.12 | 0.0030 | 1.70E-06 | 3.93E-06 |
| 3R.5943261 | *CG3940* | intron | T | C | 0.47 | 4.23E-06 | 2.99E-04 | 3.16E-06 |
| 3R.25908134 | *Sima* | Intron | C | T | 0.31 | 2.73E-06 | 0.0368 | 2.86E-04 |
| 3R.26931553 | *CG11318/CG15553* | 535/4662 | C | T | 0.04 | 5.13E-04 | 4.32E-08 | 5.34E-08 |
| 3R.26932043 | *CG11318* | dn(45) | T | G | 0.03 | 5.13E-04 | 4.32E-08 | 5.34E-08 |
| 3R.26952996 | *CG15554* | 3’UTR | G | A | 0.04 | 0.0020 | 6.90E-06 | 5.33E-06 |
| 3R.27035817 | *chp* | Intron | C | T | 0.02 | 0.1771 | 1.04E-06 | 9.55E-05 |
| X.10840192 | *Myo10a* | syn. | G | A | 0.16 | 7.88E-07 | 0.0086 | 5.35E-05 |

SNP=position in genome (chromomsome.position); gene=gene symbol ID; class=type of SNP; A1=allele 1 ID; A2=allele 2 ID; MAF=minor allele (A2) frequency; *p_high_*=*p-value* for high glucose mapping; *p_low_=p-value* for high glucose mapping; *p_pooled_*=*p-value* for mapping when both diets are combined
