# Supplementary material for: The Complex Contributions of Genetics and Nutrition to Immunity in Drosophila melanogaster
Source: PLoS Genet. 2015 Mar 12;11(3):e1005030. doi: 10.1371/journal.pgen.1005030 (PMC4357385; doi:10.1371/journal.pgen.1005030)
Supplement: S4 Table — (DOCX) [file pgen.1005030.s004.docx]

**Table S4**. Model comparisons for correlations between immune response and nutritional phenotypes when *Diptericin* state is ignored or accounted for.

| **Diet** | **Phenotype** | **F** | **P(models)** | **P(noDpt)** | **P(Dpt)** |
| --- | --- | --- | --- | --- | --- |
| High glucose | Glucose | 29.92 | <0.0001 | 0.026 | 0.078 |
|  | Glycogen | 22.85 | <0.0001 | 0.651 | 0.984 |
|  | Glycerol | 23.28 | <0.0001 | 0.11 | 0.124 |
|  | Triglyceride | 23.51 | <0.0001 | 0.913 | 0.48 |
|  | Protein | 23.3 | <0.0001 | 0.061 | 0.036 |
|  | Weight | 23.88 | <0.0001 | 0.241 | 0.104 |
| Low glucose | Glucose | 28.8 | <0.0001 | 0.619 | 0.78 |
|  | Glycogen | 28.14 | <0.0001 | 0.568 | 0.664 |
|  | Glycerol | 27.88 | <0.0001 | 0.437 | 0.598 |
|  | Triglyceride | 26.82 | <0.0001 | 0.249 | 0.572 |
|  | Protein | 27.43 | <0.0001 | 0.417 | 0.4 |
|  | Weight | 28.57 | <0.0001 | 0.273 | 0.433 |

P(models) refers to the comparison between models with and without *Diptericin* state as a covariate (in all cases, accounting for *Dpt* makes for a significantly better fit); P(noDpt) is the significance of the correlation without accounting for *Diptericin;* P(Dpt) is the significance of the correlation when accounting for *Diptericin.*
