# Supplementary figures and images for: The Complex Contributions of Genetics and Nutrition to Immunity in Drosophila melanogaster
Source: PLoS Genet. 2015 Mar 12;11(3):e1005030. doi: 10.1371/journal.pgen.1005030 (PMC4357385; doi:10.1371/journal.pgen.1005030)

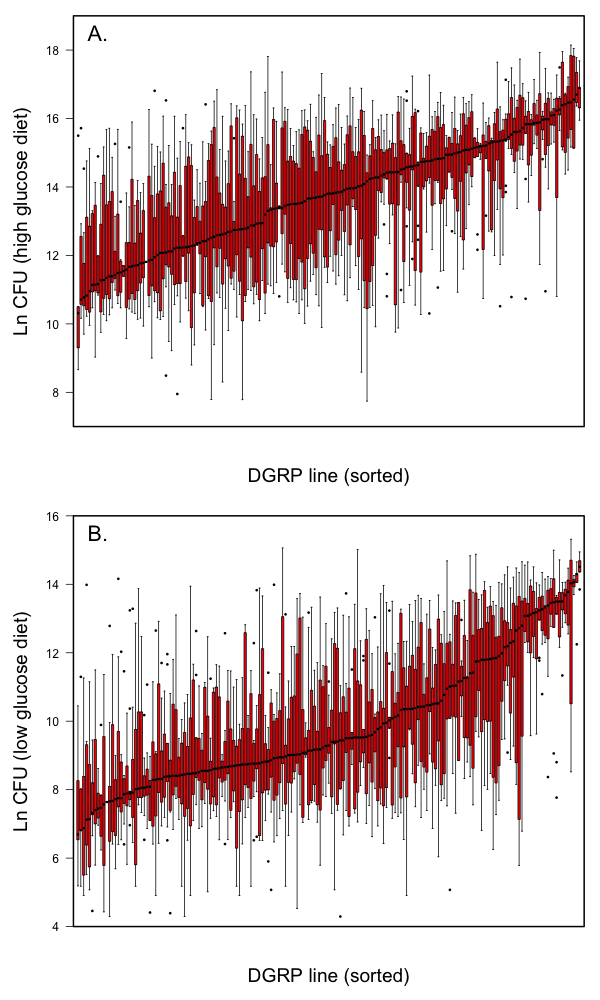

Supplement: S1 Fig — (TIFF) [file pgen.1005030.s005.tiff]

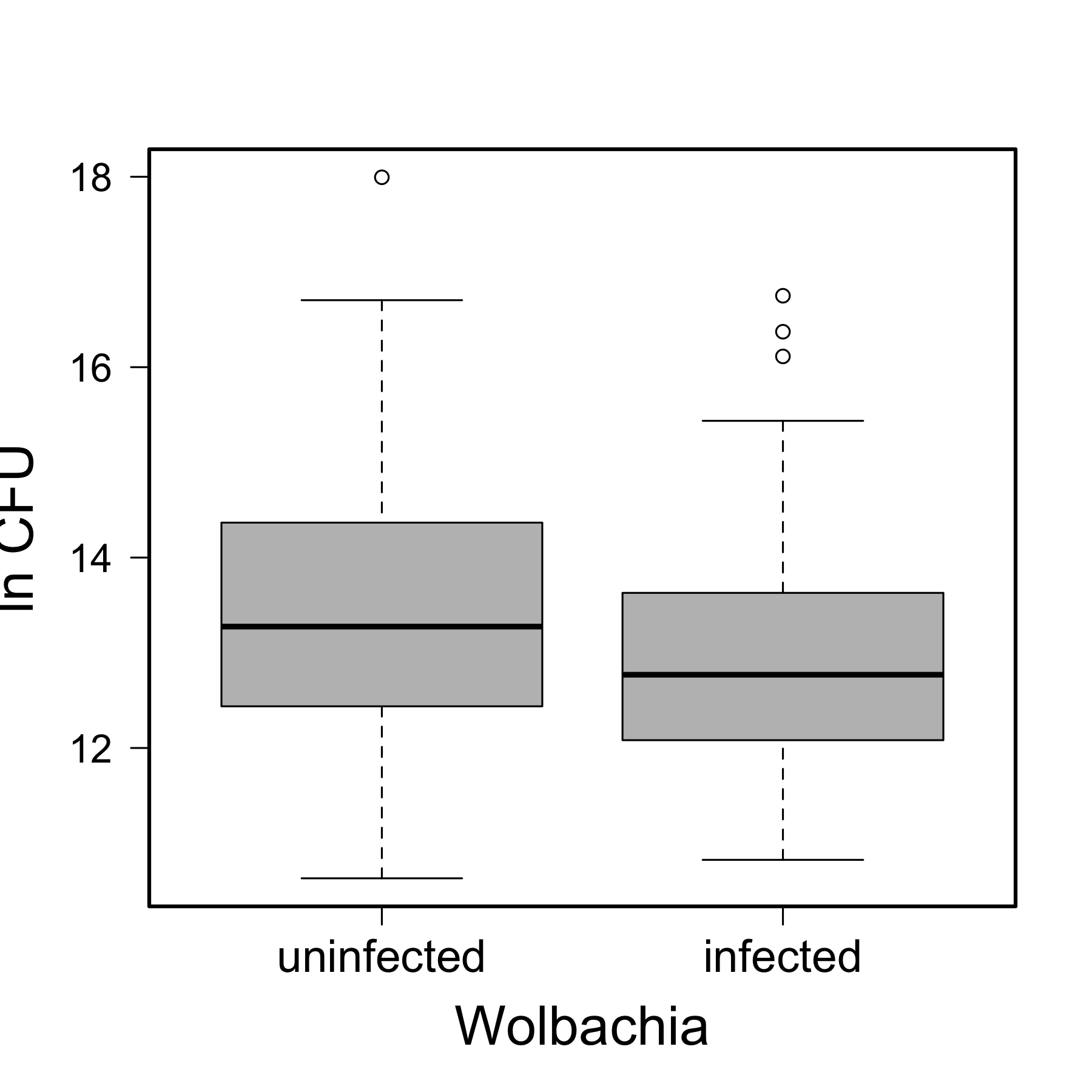

Supplement: S2 Fig — Bacterial load data is pooled across both diets. (TIFF) [file pgen.1005030.s006.tiff]

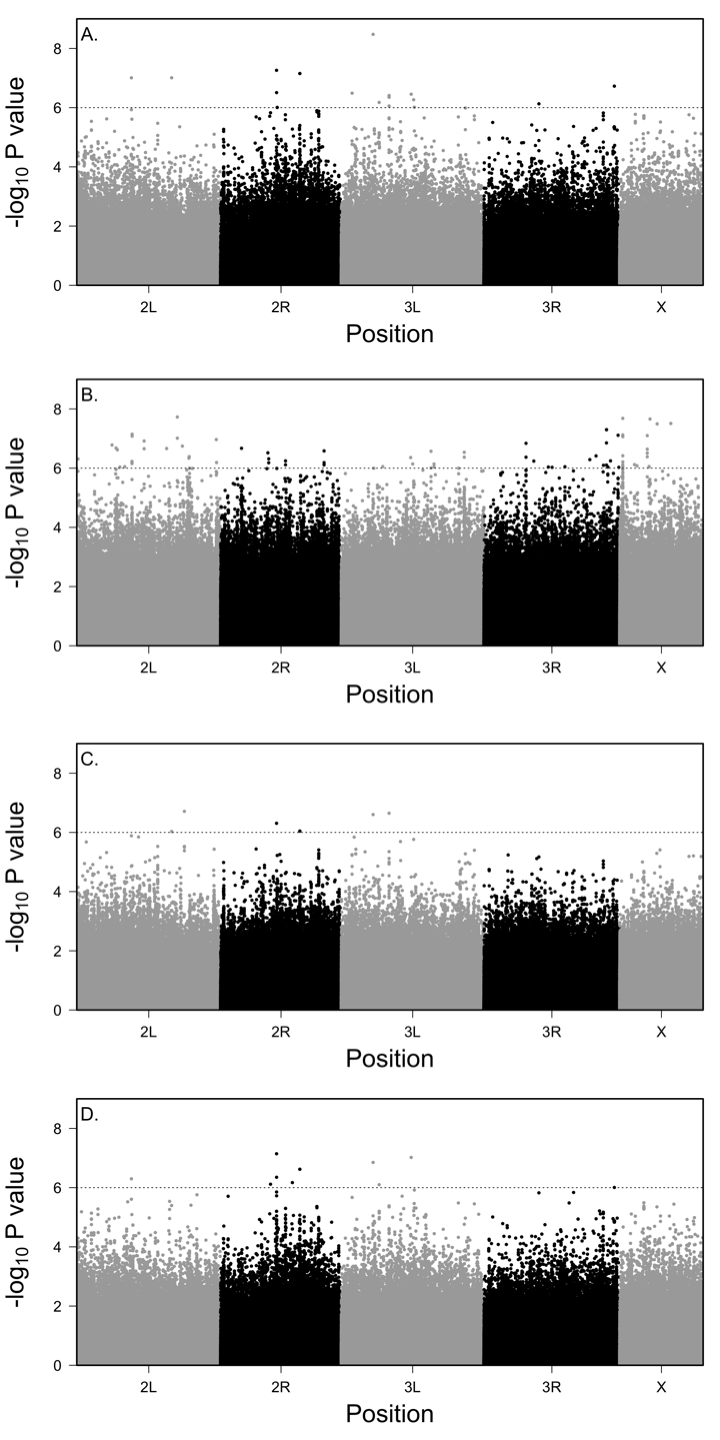

Supplement: S3 Fig — Dotted line represents nominal p-value cutoff of 10-6. (TIFF) [file pgen.1005030.s007.tiff]

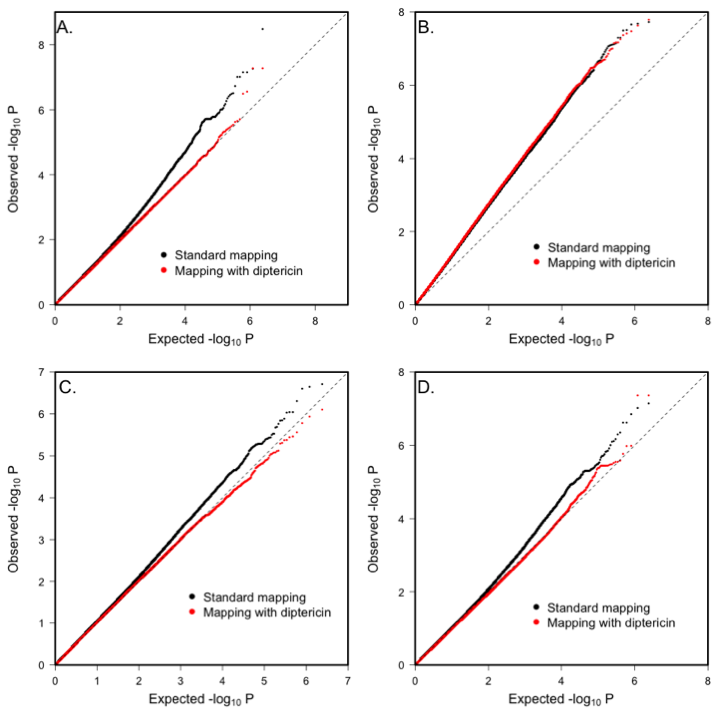

Supplement: S4 Fig — Dotted line is one to one. Black dots from standard mapping approach; red dots from mapping when Diptericin state is used as a covariate. (TIFF) [file pgen.1005030.s008.tiff]

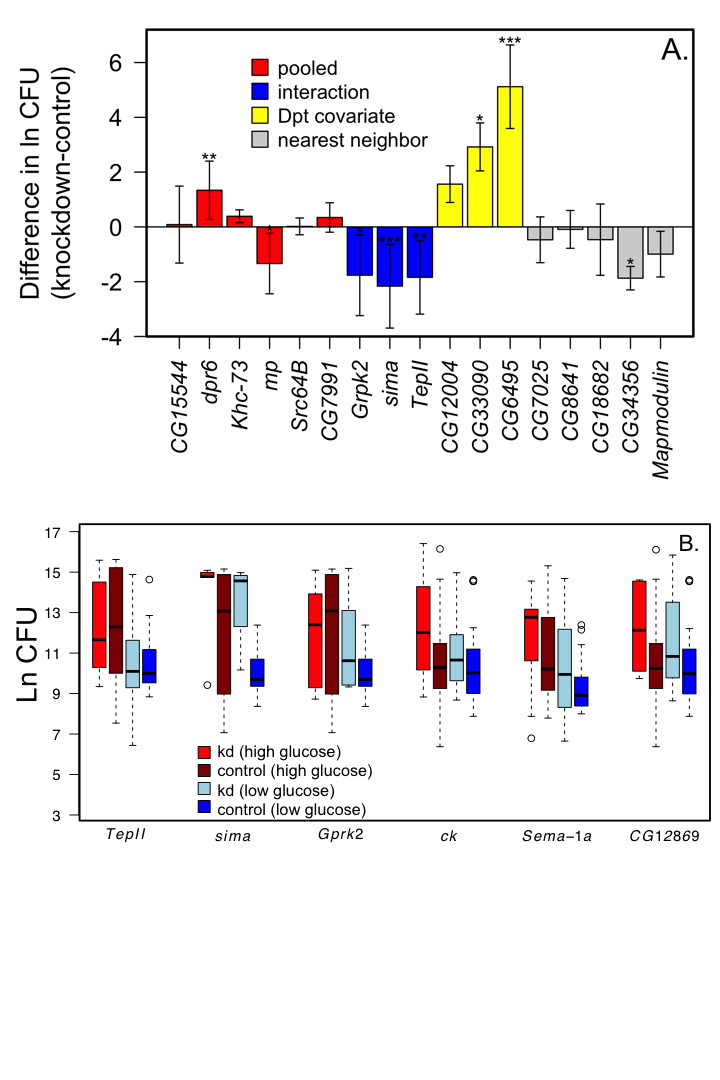

Supplement: S5 Fig — Pooled refers to genes containing SNPs that mapped when data from both diets were pooled, interaction refers to genes with SNPs that mapped for the interaction term, Dpt covariate refers to genes containing SNPs that mapped when Dpt allele was included as a covariate and nearest neighbor refers to genes selected as position matched controls. (TIFF) [file pgen.1005030.s009.tiff]

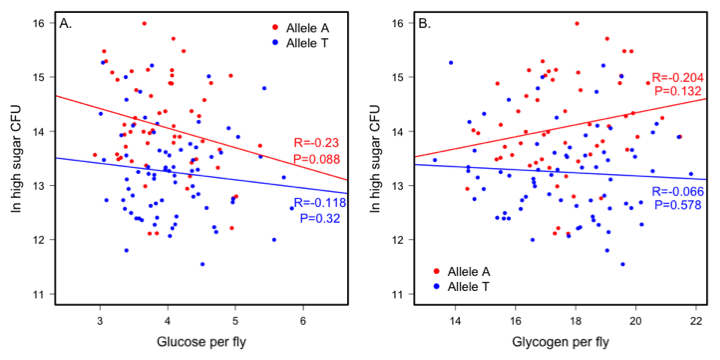

Supplement: S6 Fig — (TIFF) [file pgen.1005030.s010.tiff]

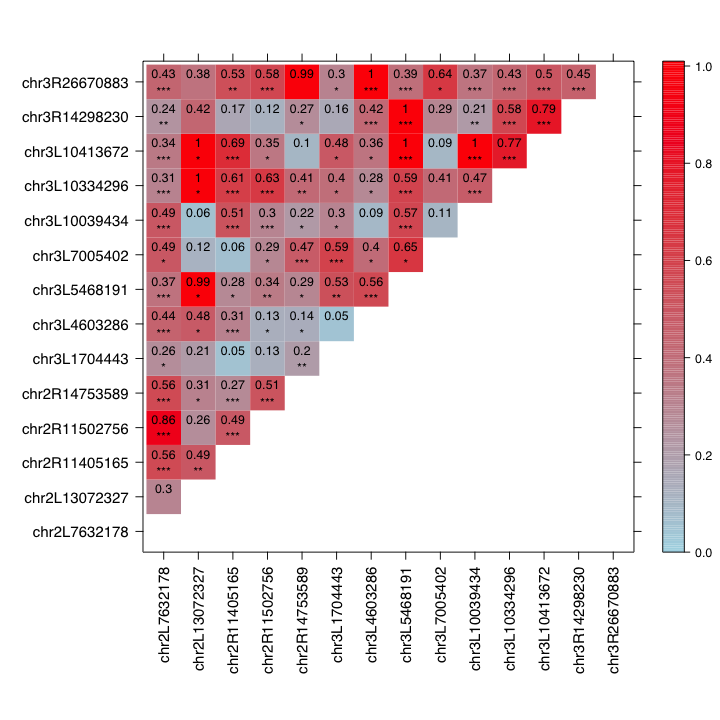

Supplement: S7 Fig — Only one SNP per gene was used to reduce signal from physical linkage. (p<0.0001***, p<0.001**, p<0.05*) (TIFF) [file pgen.1005030.s011.tiff]
